# Cortical thinning and altered directed connectivity in Parkinson’s disease: a cortical thickness–guided voxel-wise granger causality study

**DOI:** 10.3389/fneur.2026.1967317

**Published:** 2026-10-28

**Authors:** Dan Cheng, Yuxi Wang, Chengcheng Liu, Xiu Cheng, Miao Wang, Yaxin Tuo, Wen Chen, Dehong Wu

**Affiliations:** 1 Department of Radiology, Taihe Hospital, Hubei University of Medicine, Shiyan, China; 2 Department of Nuclear Medicine, Taihe Hospital, Hubei University of Medicine, Shiyan, China; 3 Department of Radiology, Guangshui First People’s Hospital, Suizhou, China

**Keywords:** cognitive function, cortical thickness, effective connectivity, granger causality analysis, Parkinson’s disease

## Abstract

**Background:**

Parkinson’s disease (PD) involves both regional cortical degeneration and large-scale network dysfunction. This study examined whether cortical regions showing structural abnormalities in PD also exhibit altered directed connectivity.

**Methods:**

Sixty-two patients with PD and 46 healthy controls underwent structural magnetic resonance imaging (MRI) and resting-state functional magnetic resonance imaging (fMRI). Whole-brain cortical thickness was analyzed using FreeSurfer. Regions showing significant cortical thinning were used as seeds for voxel-wise Granger causality analysis (GCA). Associations between imaging measures and Montreal Cognitive Assessment (MoCA) scores were examined using partial correlation analyses.

**Results:**

Patients with PD showed significantly reduced cortical thickness in the right medial superior frontal gyrus (SFGmed.R) and right precentral gyrus (PreCG.R), with no regions showing significantly greater cortical thickness than healthy controls. Compared with controls, patients with PD exhibited increased GCA from SFGmed.R to the left inferior temporal gyrus and from PreCG.R to the calcarine/medial visual cortex, whereas decreased GCA was observed from PreCG.R to the left supramarginal gyrus and from the right superior temporal gyrus and left olfactory-related region to PreCG.R. SFGmed.R cortical thickness was positively associated with MoCA scores after adjustment for age, sex, and education level, and this association remained significant after Bonferroni correction for seven exploratory correlation analyses (partial Spearman’s *ρ* = 0.847, *p* < 0.001). No other imaging–MoCA associations reached the corrected significance threshold.

**Conclusion:**

Cortical thinning in frontal-executive and motor regions of patients with PD is accompanied by altered directed interactions with distributed sensory and associative regions. Medial superior frontal cortical thickness may also be related to global cognitive performance.

## 1 Introduction

Parkinson’s disease (PD) is a progressive neurodegenerative disorder with a broad clinical spectrum. Although its clinical diagnosis remains primarily based on motor manifestations, including bradykinesia, rigidity, resting tremor, and postural instability, PD is increasingly recognized as a multisystem disorder involving both motor and non-motor domains (1). Cognitive impairment, olfactory dysfunction, sleep disturbance, affective symptoms, visual complaints, and autonomic dysfunction may occur across different stages of the disease and can substantially influence daily functioning and quality of life (2, 3). From a neuropathological perspective, PD-related α-synuclein pathology is not restricted to the nigrostriatal dopaminergic system, but may involve olfactory, brainstem, limbic, and neocortical regions during disease progression (4, 5). This clinical heterogeneity suggests that PD cannot be fully explained by nigrostriatal dopaminergic degeneration alone, but rather involves distributed cortical–subcortical circuits.

Structural magnetic resonance imaging has provided important evidence of cortical involvement in PD. Cortical thickness is a sensitive morphometric measure of cortical integrity and has been widely used to characterize neurodegenerative changes *in vivo*. Previous studies have reported cortical thinning in the frontal, temporal, parietal, occipital, and sensorimotor regions of patients with PD, particularly in association with cognitive decline and disease progression (6–10). However, the spatial distribution of cortical thickness alterations in PD varies across studies. A coordinate-based meta-analysis found limited convergence of cortical thickness abnormalities in PD, suggesting that cortical morphology alone may not fully capture disease-related neural alterations and that additional network-level information may be required to interpret regional structural findings (11).

Among cortical regions potentially involved in PD, frontal and motor cortices are of particular relevance. The medial superior frontal gyrus is involved in executive control, internally guided behavior, action monitoring, and motor planning, whereas the precentral gyrus represents a core region of the primary motor system. Alterations in these regions may therefore reflect the convergence of cognitive-executive and motor cortical vulnerability in PD. Importantly, cortical thinning in PD is unlikely to represent isolated regional damage. Recent connectome-based work suggests that neurodegeneration may be constrained by the organization of structural and functional brain networks, and that brain connectivity can help predict subsequent patterns of atrophy progression in PD (12). In addition, disrupted structural–functional coupling has been observed in PD, further supporting the view that regional cortical abnormalities should be interpreted within the context of large-scale network reorganization (13).

Resting-state functional MRI offers a complementary means of characterizing spontaneous brain network activity. In PD, altered functional connectivity has been observed across the basal ganglia, sensorimotor, default mode, salience, frontoparietal, and visual networks, and has been linked to both motor and cognitive dysfunction (14). Nevertheless, conventional functional connectivity analyses are largely based on temporal correlations between blood oxygen level-dependent (BOLD) signals and therefore reflect statistical dependence rather than the direction of interregional influence. This distinction is particularly important in PD, where disrupted communication within cortico-basal ganglia-thalamo-cortical circuits and distributed sensory-associative systems may involve not only changes in coupling strength but also abnormalities in the direction of information flow.

Effective connectivity analysis enables the investigation of directed interactions within brain networks. Granger causality analysis (GCA), originally developed for time-series prediction, assesses whether the past activity of one signal improves the prediction of the future activity of another (15). In neuroscience and neuroimaging, GCA has been applied to characterize directional relationships between neural time series. However, findings derived from fMRI-based GCA should be interpreted cautiously because of the limited temporal resolution of BOLD signals and potential hemodynamic confounds. Nevertheless, GCA may detect network abnormalities that are not apparent in conventional correlation-based analyses. In PD, this approach has revealed widespread alterations in directed functional connectivity and may be sensitive to disease-related network changes (16).

A directional network perspective may also help clarify the sensory and non-motor manifestations of PD. Visual dysfunction is common in PD and may arise from disturbances at the ocular, visuoperceptual, visuospatial, and central visual processing levels (17). Olfactory dysfunction is another frequent non-motor symptom that often occurs early in the disease course and has been proposed as a potential marker of prodromal PD (18). Alongside evidence of disrupted sensorimotor and associative network organization, these observations raise the possibility that motor cortical abnormalities are linked to dysfunction in visual, temporal, parietal, and olfactory-related systems. However, whether structurally vulnerable cortical regions also show altered directional interactions with these distributed systems remains unclear.

In the present study, we combined cortical thickness analysis with voxel-wise GCA to examine whether regional cortical abnormalities in PD are accompanied by alterations in directional brain connectivity. Cortical thickness was first compared between patients with PD and healthy controls, and regions showing significant cortical thinning were subsequently defined as data-driven seeds for whole-brain voxel-wise GCA. For each seed, both seed-to-voxel and voxel-to-seed influences were assessed and compared between groups. We hypothesized that patients with PD would exhibit cortical thinning in frontal and motor regions and that these structurally vulnerable areas would show abnormal directional interactions with distributed sensory and associative networks. Importantly, rather than examining cortical morphology and functional connectivity independently, this structure-guided approach links regional cortical vulnerability to abnormalities in directed network communication. By integrating structural and effective connectivity analyses, the present study aimed to provide a more comprehensive understanding of how focal cortical degeneration is embedded within large-scale network dysfunction in PD.

## 2 Methods

### 2.1 Participants

This cross-sectional study was conducted in accordance with the Strengthening the Reporting of Observational Studies in Epidemiology (STROBE) guidelines for observational studies (19) and was approved by the Ethics Committee of Shiyan Taihe Hospital (approval number: 2025KS54). Written informed consent was obtained from all participants before enrollment. Patients with clinically diagnosed PD were consecutively recruited from the inpatient Department of Neurology at Shiyan Taihe Hospital between December 2023 and October 2025. Healthy controls (HC) were recruited from the local community and the hospital health examination center during the same period. All patients were independently diagnosed by two senior neurologists specializing in PD and movement disorders according to the Chinese diagnostic criteria for PD formulated by the Chinese Society of Neurology (20). Healthy controls had no history of neurological or major psychiatric disorders and showed no clinically significant abnormalities on neurological examination. Participants were eligible if they had reliable demographic information, complete clinical and cognitive assessment data, and usable MRI data.

### 2.2 Clinical and demographic assessment

Demographic information, including age, sex, and education level, was collected for all participants. For patients with PD, disease duration (years) and Hoehn–Yahr (H&Y) stage were also recorded to characterize disease duration and clinical severity. Education level was coded on a four-point ordinal scale according to the highest educational attainment: 1, primary school; 2, junior high school; 3, senior high school; and 4, college or university. Cognitive function was assessed using the Montreal Cognitive Assessment (MoCA) by trained neurologists on the day of MRI scanning. The MoCA assesses multiple cognitive domains and has been shown to be sensitive for detecting mild cognitive impairment (21). The total score ranges from 0 to 30, with higher scores indicating better global cognitive performance. In accordance with the standard scoring procedure, one additional point was added for participants with education levels 1–3, corresponding to 12 or fewer years of formal education, when the unadjusted MoCA score was below 30. After education adjustment, a total score of ≥26 was considered indicative of normal cognition, whereas a score of <26 was considered indicative of cognitive impairment.

### 2.3 MRI acquisition

MRI data were acquired using a 3.0-T GE Signa Architect scanner equipped with a 48-channel phased-array head coil. The imaging protocol included high-resolution three-dimensional (3D) T1-weighted structural imaging and resting-state fMRI. High-resolution structural images were obtained using a 3D BRAVO sequence with the following parameters: repetition time/echo time (TR/TE) = 2400/2.01 ms; inversion time (TI) = 1,000 ms; flip angle (FA) = 8°; field of view (FOV) = 256 mm × 256 mm; matrix size = 256 × 256; slice thickness = 1 mm; and voxel size = 1 mm × 1 mm × 1 mm. Resting-state fMRI data were acquired using a gradient-echo echo-planar imaging sequence with the following parameters: TR/TE = 2000/30 ms; FA = 52°; 64 axial slices; slice thickness = 3 mm; voxel size = 3 mm × 3 mm × 3 mm; FOV = 240 mm × 240 mm; and matrix size = 80 × 80. During the resting-state fMRI scan, participants were instructed to keep their eyes open, remain awake, stay still, and let their minds wander without engaging in any specific cognitive task.

### 2.4 Imaging data preprocessing

Structural T1-weighted images were processed using FreeSurfer 7.2.0 to estimate cortical thickness (22, 23). The standard FreeSurfer recon-all pipeline was applied, including intensity non-uniformity correction, skull stripping, Talairach transformation, segmentation of subcortical white matter and deep gray matter structures, tessellation of the gray–white matter boundary, topology correction, surface deformation, and reconstruction of the white and pial surfaces. Cortical thickness was calculated as the distance between the white matter surface and the pial surface at each vertex across the cortical mantle. All reconstructed cortical surfaces were visually inspected to ensure processing quality. Group comparisons of cortical thickness between PD and HC were then performed to identify regions showing significant cortical thickness alterations in PD. Brain regions with significant between-group differences in cortical thickness were defined as seed regions for subsequent effective connectivity analysis.

Resting-state fMRI data were preprocessed using RESTplus based on Statistical Parametric Mapping 12 (SPM12). The first ten volumes of each functional time series were discarded to allow for signal equilibrium and participant adaptation to the scanning environment. The remaining images were corrected for slice timing and realigned for head motion correction. Participants with excessive head motion were excluded according to predefined motion criteria (24). Functional images were then spatially normalized to the Montreal Neurological Institute (MNI) space and resampled to 3 × 3 × 3 mm^3^ voxels using standard SPM-based normalization procedures. Nuisance covariates, including head motion parameters, white matter signals, cerebrospinal fluid signals, and linear trends, were regressed out to reduce non-neuronal effects. Spatial smoothing was performed using a 6-mm full width at half maximum (FWHM) Gaussian kernel, followed by temporal band-pass filtering at 0.01–0.1 Hz (25). Based on the structurally defined seed regions derived from the cortical thickness analysis, voxel-wise GCA was conducted using RESTplus. For each seed region, both seed-to-voxel and voxel-to-seed GCA maps were generated to characterize directional effective connectivity, and these maps were subsequently entered into group-level statistical analyses to compare PD and HC.

### 2.5 Cortical thickness analysis

Whole-brain cortical thickness analysis was performed using FreeSurfer 7.2.0. Cortical thickness was calculated at each vertex as the shortest distance between the gray–white matter boundary and the pial surface, providing a vertex-wise measure of cortical morphometry across the cerebral cortex (26). After surface reconstruction and quality inspection, individual cortical thickness maps were registered to a common surface-based template to enable group-level comparison.

Between-group differences in cortical thickness between patients with Parkinson’s disease and healthy controls were assessed using a general linear model. Age, sex, and education level were included as nuisance covariates to reduce the potential influence of demographic variables on cortical thickness. Multiple comparisons across the cortical surface were corrected using cluster-level family-wise error (FWE) correction. A vertex-level threshold of *p* < 0.001, uncorrected, was first applied to define clusters, and clusters surviving a cluster-level FWE-corrected threshold of *p* < 0.05 were considered statistically significant. Cortical clusters showing significant between-group differences were defined as structurally abnormal cortical regions in PD and were subsequently used as seed regions for voxel-wise Granger causality analysis.

### 2.6 Voxel-wise GCA

Voxel-wise GCA was performed using RESTplus to investigate directed effective connectivity associated with the cortical regions showing significant thickness alterations. Each cluster exhibiting significant cortical thickness differences between PD and HC was defined as a seed region (27). For each seed, two directional GCA maps were generated, including seed-to-voxel and voxel-to-seed analyses (28). The seed-to-voxel analysis quantified the directional influence from the seed region to all other brain voxels, whereas the voxel-to-seed analysis quantified the directional influence from all brain voxels to the seed region. The resulting GCA coefficients were transformed into standardized maps and entered into group-level analyses. Between-group comparisons were subsequently performed to identify alterations in directed effective connectivity associated with structurally vulnerable cortical regions in PD (29). GCA was selected because the primary aim of the present study was to perform an exploratory, structure-guided, whole-brain assessment of directional temporal predictability between cortical regions identified by morphometric analysis and the rest of the brain. The voxel-wise GCA framework allows bidirectional seed-to-voxel and voxel-to-seed mapping without requiring the specification and fitting of an explicit whole-brain generative model, and was therefore well suited to our data-driven seed-selection strategy. This methodological choice was not intended to imply superiority over other approaches to directed or effective connectivity, which may provide complementary information.

### 2.7 Statistical analyses

Statistical analyses for demographic and clinical data were performed using SPSS version 23.0, with the significance level set at *p* < 0.05. The Shapiro–Wilk test was used to assess the normality of continuous variables. Normally distributed variables were expressed as mean ± standard deviation and compared between the PD and HC groups using independent two-sample t-tests. Non-normally distributed variables were expressed as median (25th–75th percentiles) and compared between the PD and HC groups using the Mann–Whitney U test. Categorical variables, including sex, were analyzed using the chi-square test or Fisher’s exact test, as appropriate. Disease duration and H&Y stage were reported descriptively for the PD group only and were not included in between-group comparisons. Disease duration was summarized as the median (25th–75th percentiles) because of its non-normal distribution, whereas H&Y stage, as an ordinal variable, was also summarized as the median (25th–75th percentiles).

For cortical thickness analysis, vertex-wise general linear model analyses were performed using FreeSurfer to compare cortical thickness between the PD and HC groups. Age, sex, and education level were included as covariates. Multiple comparisons across the cortical surface were corrected using cluster-level family-wise error correction. The statistical threshold was set at a vertex-level *p* < 0.001, uncorrected, and a cluster-level FWE-corrected *p* < 0.05. Cortical clusters showing significant between-group differences were defined as structurally abnormal seed regions and were subsequently used for voxel-wise Granger causality analysis.

For voxel-wise GCA, group-level two-sample t-tests were performed on the standardized GCA coefficient maps using RESTplus/SPM12. For each structurally defined seed region, both seed-to-voxel and voxel-to-seed GCA maps were analyzed separately. Age, sex, education level, and mean framewise displacement were included as covariates to control for potential demographic and head-motion effects. To control for multiple comparisons, the significance threshold for between-group comparisons was set at a voxel-level *p* < 0.001, uncorrected, and a cluster-level Gaussian random field (GRF)-corrected *p* < 0.05, two-tailed. Significant clusters were reported with anatomical location, peak Montreal Neurological Institute coordinates, peak *T* value, cluster size, and corrected *p* value.

To further examine whether altered cortical thickness and directed effective connectivity were associated with cognitive performance in patients with PD, exploratory partial correlation analyses were performed between the imaging measures and MoCA scores. Mean cortical thickness values were extracted from cortical clusters showing significant between-group differences, and mean GCA values were extracted from clusters showing significant group differences. Partial Pearson’s correlation analysis was used when the variables were normally distributed, whereas partial Spearman’s rank correlation analysis was used when the normality assumption was not met. Age, sex, and education level were included as covariates in all correlation analyses. To account for multiple testing across the seven exploratory imaging–MoCA correlation analyses, Bonferroni correction was applied, resulting in a corrected significance threshold of *p* < 0.0071 (0.05/7).

## 3 Results

### 3.1 Demographic and clinical characteristics

As summarized in Table 1, a total of 108 participants were included in the study, comprising 62 patients with Parkinson’s disease (PD) and 46 healthy controls (HC). There were no statistically significant between-group differences in sex distribution (PD: 32 males and 30 females; HC: 26 males and 20 females; *χ*^2^ = 0.256, *p* = 0.698), age (PD: 63.2 ± 8.2 years; HC: 61.0 ± 3.9 years; t = 1.692, *p* = 0.094), or education level (PD: 3 (2–4); HC: 3 (2–4); Z = −0.307, *p* = 0.759). Patients with PD had significantly lower MoCA scores than healthy controls (20.5 (12–23) vs. 27 (27–28); Z = −7.699, *p* < 0.001), indicating poorer global cognitive performance in the PD group. The median disease duration was 2.5 (2–5) years, and the median H&Y stage was 1.5 (1–2) in the PD group.

**Table 1:** Demographic and clinical characteristics of the participants.

Characteristics	PD	HC	Statistics	*p*-value
Case	62	46	N/A	N/A
Sex (M/F)	32/30	26/20	*χ*^2^ = 0.256	0.698
Age (years old)	63.2 ± 8.2	61.0 ± 3.9	T = 1.692	0.094
Education level	3 (2–4)	3 (2–4)	Z = −0.307	0.759
MOCA scores	20.5 (12–23)	27 (27–28)	Z = −7.699	<0.001
Disease duration (years)	2.5 (2–5)	N/A	N/A	N/A
H&Y	1.5 (1–2)	N/A	N/A	N/A

Data are presented as number, mean ± standard deviation (SD), or median (25th, 75th percentiles), as appropriate. Education level was coded on a four-point ordinal scale according to the highest educational attainment: 1, primary school; 2, junior high school; 3, senior high school; and 4, college or university. Abbreviations: PD, Parkinson’s disease; HC, healthy controls; MoCA, Montreal Cognitive Assessment; H&Y, Hoehn–Yahr stage; M, male; F, female; N/A, not applicable. a: *χ*^2^-test. b: Two-sample *t*-test. c: Mann–Whitney U test.

### 3.2 Cortical thickness alterations

As shown in Figure 1 and Table 2, whole-brain vertex-wise cortical thickness analysis identified significant cortical thinning in the PD group compared with the HC group. Statistical significance was determined using a vertex-level threshold of *p* < 0.001 uncorrected and a cluster-level corrected threshold of *p* < 0.05. After adjusting for age, sex, and education level, patients with PD exhibited significantly reduced cortical thickness in the right medial superior frontal gyrus (SFGmed.R) and the right precentral gyrus (PreCG.R). No clusters showed significantly greater cortical thickness in PD patients relative to healthy controls. Based on these findings, the right medial superior frontal gyrus and right precentral gyrus were selected as structurally abnormal seed regions for subsequent voxel-wise Granger causality analysis.

**Figure 1:**
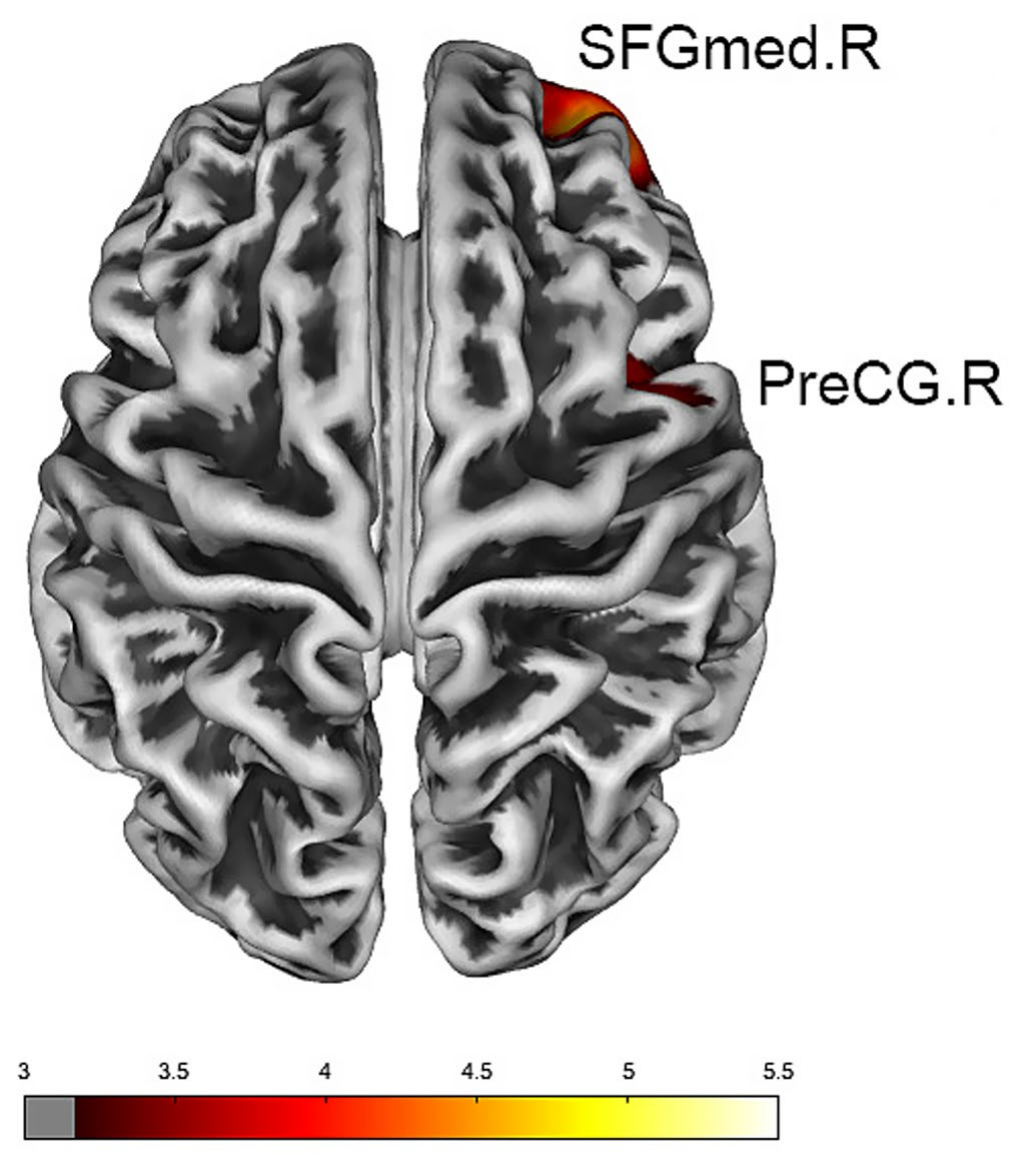
Group differences in cortical thickness between patients with Parkinson’s disease (PD) and healthy controls (HCs). Only brain views showing significant between-group differences are displayed. Whole-brain vertex-wise analysis revealed significantly reduced cortical thickness in the right medial superior frontal gyrus (SFGmed.R) and right precentral gyrus (PreCG.R) in patients with PD compared with HCs. The significant clusters are directly labeled in the figure.

**Table 2:** Brain regions showing significant cortical thinning in patients with Parkinson’s disease.

Contrast	Brain region	Peak MNI (x, y, z)	Peak stat	Cluster size	Cluster-level pFWE
HC > PD	SFGmed.R	22, 45, 1	4.75	382	<0.001
HC > PD	PreCG.R	38, −2, 34	3.70	202	0.012

PD, Parkinson’s disease; HC, healthy control; SFGmed.R, right medial superior frontal gyrus; PreCG.R, right precentral gyrus; MNI, Montreal Neurological Institute; FWE, family-wise error.

### 3.3 Voxel-wise granger causality analysis

#### 3.3.1 Seed-to-voxel GCA of the SFGmed.R

The seed-to-voxel GCA analysis using the SFGmed.R seed revealed a significant group difference in the left inferior temporal gyrus (ITG.L) (Table 3, Figure 2A). Compared with the HC group, the PD group exhibited significantly increased GCA from SFGmed.R to ITG.L. This cluster survived GRF correction at a two-tailed threshold of *p* < 0.05.

**Table 3:** Group differences in voxel-wise Granger causality based on structurally abnormal seed regions.

Seed region	Direction	Contrast	Brain region	Peak MNI (x, y, z)	Peak T	Cluster size	Corrected *p*-value
SFGmed.R	Seed-to-voxel	PD > HC	ITG.L	−42, −45, −18	4.9316	1,417	<0.05
PreCG.R	Seed-to-voxel	PD > HC	CAL.L	3, −84, 9	3.6210	426	<0.05
PreCG.R	Seed-to-voxel	PD < HC	SMG.L	−21, −45, 39	−4.2245	750	<0.05
PreCG.R	Voxel-to-seed	PD < HC	STG.R	39, 12, −18	−3.7552	637	<0.05
PreCG.R	Voxel-to-seed	PD < HC	OLF.L	−21, 3, −15	−4.1893	505	<0.05

PD, Parkinson’s disease; HC, healthy control; GCA, Granger causality analysis; SFGmed.R, right medial superior frontal gyrus; PreCG.R, right precentral gyrus; ITG.L, left inferior temporal gyrus; CAL.L, left calcarine cortex; SMG.L, left supramarginal gyrus; STG.R, right superior temporal gyrus; OLF.L, left olfactory cortex; MNI, Montreal Neurological Institute.

**Figure 2:**
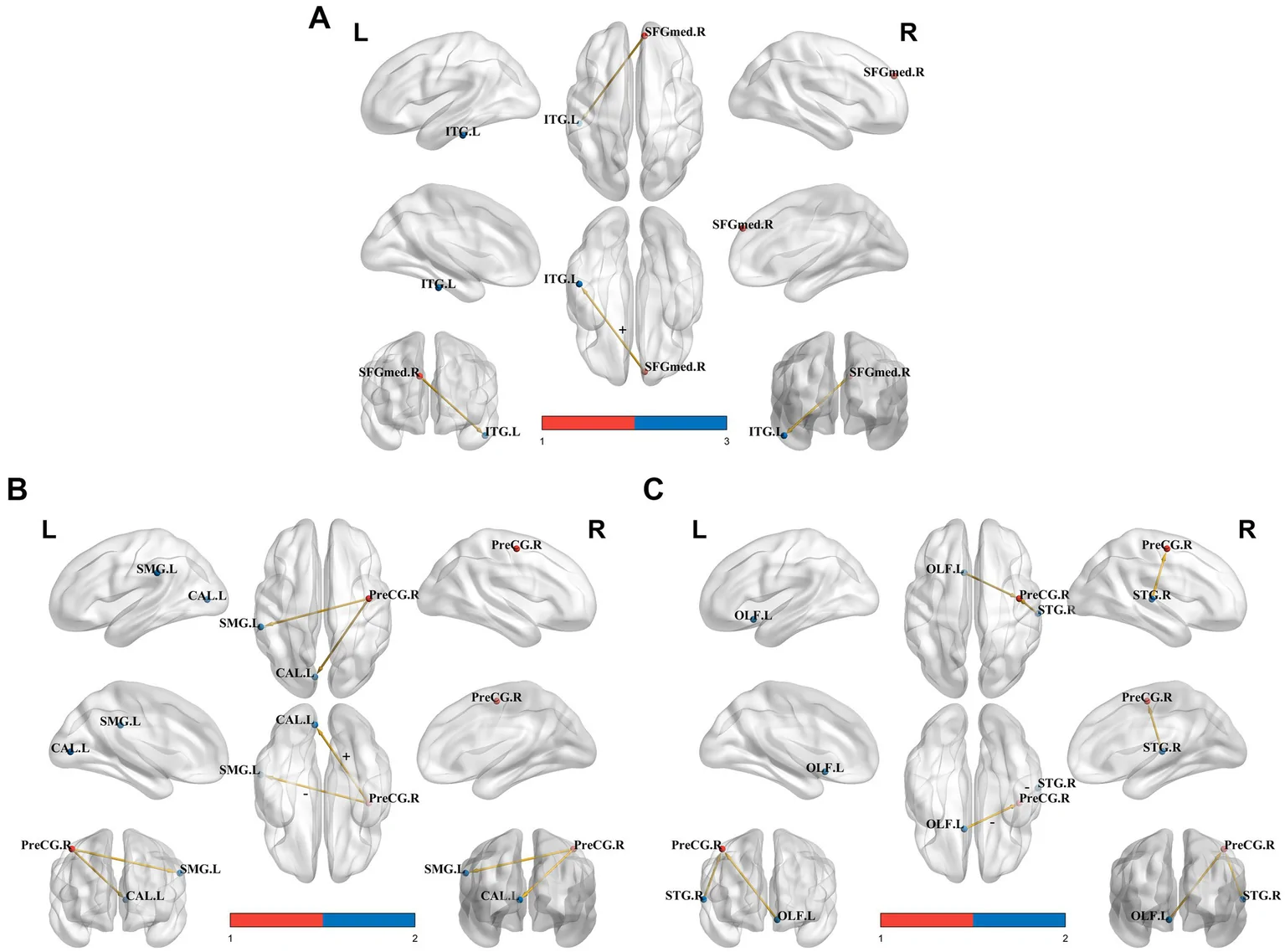
**(A)** Group differences in effective connectivity based on the structurally abnormal seed region. Seed-to-voxel Granger causality analysis using the right medial superior frontal gyrus (SFGmed.R) as the seed revealed significantly increased effective connectivity from SFGmed.R to the left inferior temporal gyrus (ITG.L) in patients with PD compared with HCs. Arrows indicate the direction of effective connectivity, and the “+” indicates increased GCA in the PD group. The cluster survived Gaussian random field correction at a two-tailed threshold of *p* < 0.05. **(B)** Group differences in effective connectivity based on the structurally abnormal seed region. Seed-to-voxel Granger causality analysis using the right precentral gyrus (PreCG.R) as the seed revealed significantly increased effective connectivity from PreCG.R to the left calcarine cortex/medial visual cortex (CAL.L) and significantly decreased effective connectivity from PreCG.R to the left supramarginal gyrus (SMG.L) in patients with PD compared with HCs. Arrows indicate the direction of effective connectivity, the “+” indicates increased GCA, and the “−” indicates decreased GCA in the PD group. Both clusters survived Gaussian random field correction at a two-tailed threshold of *p* < 0.05. **(C)** Group differences in effective connectivity based on the structurally abnormal seed region. Voxel-to-seed Granger causality analysis using the right precentral gyrus (PreCG.R) as the seed revealed significantly decreased effective connectivity from the right superior temporal gyrus (STG.R) and the left olfactory-related region (OLF.L) to PreCG.R in patients with PD compared with HCs. Arrows indicate the direction of effective connectivity, and the “−” indicates decreased GCA in the PD group. Both clusters survived Gaussian random field correction at a two-tailed threshold of *p* < 0.05.

#### 3.3.2 Seed-to-voxel GCA of the PreCG.R

The seed-to-voxel GCA analysis using the PreCG.R seed revealed significant group differences in the calcarine cortex/medial visual cortex and the left supramarginal gyrus (SMG.L) (Table 3, Figure 2B). Compared with the HC group, the PD group showed significantly increased GCA from PreCG.R to the calcarine cortex/medial visual cortex, whereas decreased GCA from PreCG.R to SMG.L was observed. Both clusters survived GRF correction at a two-tailed threshold of *p* < 0.05.

#### 3.3.3 Voxel-to-seed GCA of the PreCG.R

The voxel-to-seed GCA analysis using the PreCG.R seed revealed significant group differences in the right superior temporal gyrus (STG.R) and the left olfactory-related region (OLF.L) (Table 3, Figure 2C). Compared with the HC group, the PD group exhibited significantly decreased GCA from STG.R and OLF.L to PreCG.R. Both clusters survived GRF correction at a two-tailed threshold of *p* < 0.05.

#### 3.3.4 Association with clinical symptoms

After Bonferroni correction for seven exploratory imaging–MoCA correlation analyses (corrected significance threshold, *p* < 0.0071), partial Spearman’s rank correlation analysis revealed a significant positive association between cortical thickness in the right medial superior frontal gyrus (SFGmed.R) and MoCA scores in the PD group after controlling for age, sex, and education level (partial Spearman’s *ρ* = 0.847, *p* < 0.001; Figure 3). Greater cortical thickness in the SFGmed.R was associated with better global cognitive performance. No other associations reached the Bonferroni-corrected significance threshold. Greater cortical thickness in the SFGmed.R was associated with better global cognitive performance. No significant associations were observed between MoCA scores and cortical thickness in the right precentral gyrus or the altered GCA measures.

**Figure 3:**
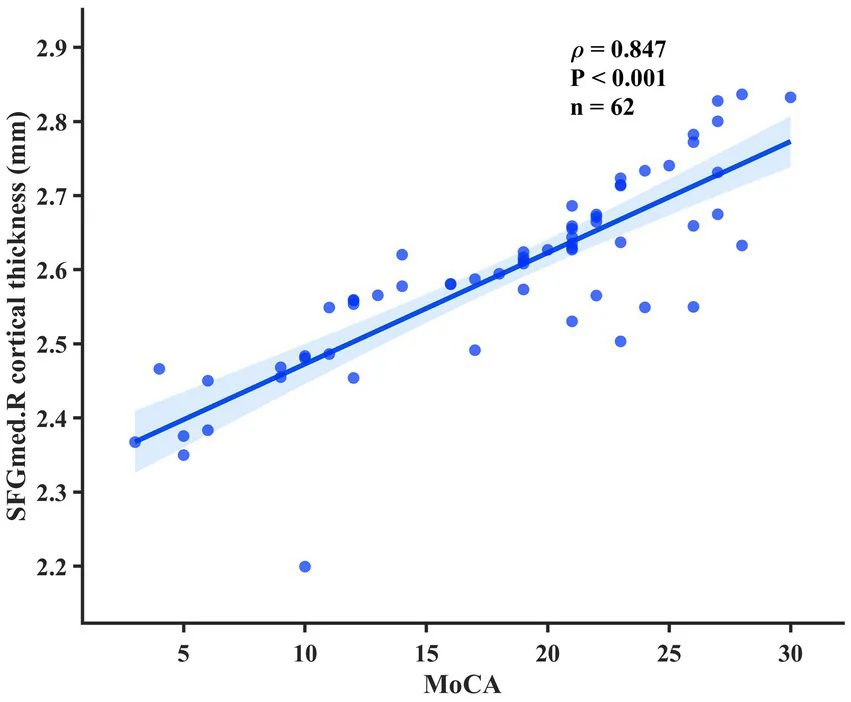
Association between cortical thickness in the right medial superior frontal gyrus and MoCA scores in patients with Parkinson’s disease. Each point represents one patient with PD. The solid line and shaded area indicate the linear trend and its 95% confidence interval based on the raw data. Partial Spearman’s rank correlation analysis revealed a significant positive association between cortical thickness in the Right medial superior frontal gyrus (SFGmed.R) and MoCA scores after controlling for age, sex, and education level (partial Spearman’s *ρ* = 0.847, *p* < 0.001; *n* = 62). The association remained significant after Bonferroni correction for seven exploratory correlation analyses (corrected significance threshold, *p* < 0.0071). SFGmed.R, Right medial superior frontal gyrus; MoCA, Montreal Cognitive Assessment; PD, Parkinson’s disease.

## 4 Discussion

In the present study, we combined cortical thickness analysis with voxel-wise Granger causality analysis to investigate whether structurally abnormal cortical regions in Parkinson’s disease exhibit altered directed effective connectivity. The main findings were as follows. First, compared with healthy controls, patients with PD showed significantly reduced cortical thickness in the right medial superior frontal gyrus and the right precentral gyrus. Second, these structurally abnormal regions showed altered directional effective connectivity with distributed temporal, visual, parietal, and olfactory-related regions. Specifically, PD patients exhibited increased GCA from SFGmed.R to ITG.L and from PreCG.R to the calcarine cortex/medial visual cortex, but decreased GCA from PreCG.R to SMG.L and from STG.R/OLF.L to PreCG.R. Third, cortical thickness in the SFGmed.R was significantly and positively associated with MoCA scores in the PD group after controlling for age, sex, and education level, whereas no significant associations were observed between MoCA scores and cortical thickness in the PreCG.R or the altered GCA values. Together, these findings suggest that cortical thinning in PD is embedded within broader abnormalities of directional brain network communication, while structural integrity of the medial superior frontal cortex may also be related to global cognitive performance. The scientific contribution of the present study lies in the integration of cortical morphometry with direction-sensitive effective connectivity analysis. First, we identified structurally vulnerable cortical regions using whole-brain cortical thickness analysis rather than selecting seed regions *a priori*. Second, these structurally defined regions were subsequently used to characterize bidirectional voxel-wise GCA patterns, allowing us to examine how focal cortical abnormalities are embedded within distributed network dysfunction. Third, the identified directional alterations involved motor, visual, temporal, parietal, and olfactory-related systems, suggesting that cortical vulnerability in PD may be associated with broader multisystem network reorganization. Finally, the association between SFGmed.R cortical thickness and MoCA scores provides preliminary evidence linking structural integrity of a frontal cortical region to global cognitive performance. Together, these findings extend previous studies that have examined cortical morphology or functional connectivity separately and provide a structure-guided framework for investigating directional network abnormalities in PD.

Our findings of cortical structural alterations in frontal regions further support the view that PD involves brain changes beyond the classical nigrostriatal dopaminergic system, which aligns with the current recognition of PD as a multisystem disorder affecting both motor and non-motor domains (30–34). More recent studies further indicate that the spatial distribution of cortical alterations in PD is heterogeneous and may be influenced by clinical and demographic characteristics. In a large multisite study of non-demented patients with PD, Oltra et al. demonstrated sex-related differences in cortical thickness involving superior frontal and precentral regions, with the extent of cortical thinning also related to age and disease duration (35). Previous structural MRI studies have reported cortical thinning in frontal, temporal, parietal, occipital, and sensorimotor regions in patients with PD, particularly in relation to cognitive decline and disease progression (36–38). Notably, Sokołowski et al. (37) reported longitudinal cortical thickness changes in PD and found that changes in cortical thickness in the inferior temporal and precentral gyri were associated with changes in cognitive performance. These findings show partial regional convergence with the present involvement of the SFGmed.R and PreCG.R, while also indicating that the anatomical distribution of cortical abnormalities varies considerably across PD cohorts. However, the spatial distribution of cortical thickness alterations in PD remains inconsistent, and a coordinate-based meta-analysis reported limited convergence of cortical thickness abnormalities across studies (11). The heterogeneity of cognitive phenotypes in PD may further contribute to variation in the anatomical patterns observed across cohorts (39). Such differences may reflect variation in disease stage, cognitive status, sex distribution, disease duration, medication exposure, sample characteristics, imaging pipelines, and statistical thresholds. Thus, the relatively focal cortical thinning observed in the present study should be interpreted as the most robust structural abnormalities detected in our cohort rather than as evidence that cortical degeneration in PD is restricted to the SFGmed.R and PreCG.R. Notably, both cortical thinning clusters were located in the right hemisphere. Because motor symptom laterality and side of disease onset were not examined, it remains unclear whether this right-lateralized pattern reflects disease-related asymmetry or sample-specific variability.

Importantly, cortical thinning in PD should not be interpreted only as a local structural abnormality. Recent connectome-based evidence suggests that neurodegeneration may be constrained by the organization of structural and functional brain networks, and brain connectivity may help predict patterns of atrophy progression in PD (40). In addition, disrupted structural–functional coupling has been reported in PD, further supporting the view that regional cortical abnormalities should be interpreted within the context of large-scale network reorganization (41, 42). Resting-state functional connectivity studies have also demonstrated widespread network abnormalities in PD, involving sensorimotor, basal ganglia, default mode, salience, frontoparietal, and visual-related networks (43–45). Previous directed-connectivity studies have further demonstrated abnormalities in the direction of information flow in PD. Goelman et al. (46) reported altered sensorimotor directed connectivity in patients with PD, whereas Mellema et al. (47) showed that causal-connectivity features involving motor regions, deep nuclei, and the thalamus were associated with subsequent motor and cognitive outcomes. More recently, Shan et al. (48) applied voxel-wise GCA in patients with PD and probable rapid eye movement sleep behavior disorder and identified reduced effective connectivity from the right precentral gyrus to the right caudate nucleus. Although the patient populations and specific target regions differ from those in the present study, both studies implicate the precentral cortex as an important node of abnormal directional communication in PD. In the present study, the PreCG.R showed altered outgoing connectivity with visual and parietal regions and reduced incoming connectivity from temporal and olfactory-related regions. Thus, our findings complement previous evidence of abnormal motor–subcortical effective connectivity by suggesting that precentral cortical dysfunction may also involve distributed sensory and associative systems. Compared with conventional functional connectivity, GCA provides information about directional predictability between time series, although fMRI-based GCA should be interpreted cautiously because of the temporal resolution of BOLD signals and possible hemodynamic confounds (49). Therefore, using cortical thickness-defined abnormal regions as seed regions for voxel-wise GCA may provide additional information about how local cortical structural abnormalities are embedded within broader directed network alterations. The choice of GCA should also be considered in relation to alternative approaches to directed or effective connectivity. Transfer entropy (TE) provides an information-theoretic measure of directed information transfer and can capture nonlinear dependencies that may not be fully represented by conventional linear GCA. Notably, Granger causality and transfer entropy are theoretically equivalent for Gaussian variables, while TE provides a broader framework for characterizing nonlinear interactions (50). MOU-EC represents a different, model-based approach in which a multivariate Ornstein–Uhlenbeck generative model is fitted to the spatiotemporal covariance structure of BOLD signals to estimate directed network interactions (51). Such a framework also enables computational investigation of how activity propagates through the estimated network. For example, Panda et al. used MOU-EC together with in silico perturbational analyses to characterize impaired integration and broadcasting of information in disorders of consciousness (52). Although that study addressed a different neurological condition, it illustrates how generative whole-brain modeling can provide mechanistic information complementary to data-driven measures of directional connectivity. Applied to PD, similar model-based analyses could help determine how alterations involving the SFGmed.R and PreCG.R influence the propagation of activity across distributed motor, sensory, and associative networks. Future studies combining GCA with TE, MOU-EC, or related generative approaches may therefore help assess the robustness of the present findings across methodological frameworks and provide additional mechanistic insight into PD-related network dysfunction.

For the SFGmed.R seed, PD patients showed increased GCA from SFGmed.R to the left inferior temporal gyrus. The medial superior frontal gyrus is involved in executive control, internally guided behavior, action monitoring, and motor planning, whereas the inferior temporal gyrus is associated with higher-order visual processing, object recognition, and semantic-related functions. Higher GCA coefficients from SFGmed.R to ITG.L may indicate altered directional interactions between frontal-executive and temporal associative regions in PD. However, because the present study did not include domain-specific executive, semantic, or visual assessments, the functional significance of this alteration remains uncertain. The increased GCA may reflect compensatory recruitment, maladaptive overcoupling, altered network excitability, or other forms of network reorganization and should therefore not be interpreted as definite functional compensation.

For the PreCG.R seed, seed-to-voxel analysis revealed increased GCA from PreCG.R to the calcarine cortex/medial visual cortex. The calcarine cortex is a primary visual processing region, and visual dysfunction is increasingly recognized as an important non-motor feature of PD (53). Enhanced directed influence from the motor cortex to medial visual regions may reflect altered visuomotor integration in PD. External visual cueing can facilitate gait performance in some patients with PD (54), suggesting that interactions between visual and motor systems may be clinically relevant. Nevertheless, because visual function, gait performance, and cueing responses were not assessed in the present study, the observed PreCG.R-to-calcarine alteration cannot be directly attributed to compensatory visuomotor mechanisms. Rather, this finding suggests that motor cortical abnormalities in PD may be associated with altered directional communication with visual-processing systems.

In contrast, PD patients showed decreased GCA from PreCG.R to the left supramarginal gyrus. The supramarginal gyrus, as part of the inferior parietal lobule, is involved in sensorimotor integration, body representation, attentional processing, and action-related functions. Reduced directed influence from PreCG.R to SMG.L may indicate weakened motor-to-parietal communication, potentially reflecting impaired integration between motor output and sensory-associative processing. This finding is relevant to PD because effective motor control depends not only on primary motor execution but also on the continuous integration of somatosensory feedback, spatial information, and action monitoring. Therefore, decreased PreCG.R → SMG.L GCA may reflect disrupted motor-parietal information flow in PD.

The voxel-to-seed analysis further showed decreased GCA from STG.R and OLF.L to PreCG.R. The superior temporal gyrus is involved in auditory, language-related, social perceptual, and higher-order sensory processing, whereas olfactory-related regions are relevant to one of the earliest and most common non-motor manifestations of PD (55, 56). Recent resting-state fMRI evidence has also demonstrated abnormalities in olfactory-related functional networks in PD. In particular, Cieri et al. (57) reported reduced functional connectivity within olfactory-related networks in PD, with greater disruption observed in patients with cognitive impairment. These findings support the broader involvement of olfactory networks in PD-related functional dysconnectivity. However, the present finding provides different information because reduced OLF.L → PreCG.R GCA reflects altered directional predictability from an olfactory-related region toward the motor cortex rather than conventional undirected functional connectivity. Lower GCA coefficients from STG.R and OLF.L may therefore reflect altered directional interactions between motor and sensory-associative systems in PD. This pattern supports the view that motor cortical dysfunction in PD may be embedded within broader multisensory and non-motor network disturbances rather than being restricted to classical motor circuits.

The significant positive association between SFGmed.R cortical thickness and MoCA scores suggests that greater structural integrity of the medial superior frontal cortex may be related to better global cognitive performance in patients with PD. The medial superior frontal gyrus contributes to executive control, internally guided behavior, action monitoring, and motor planning (58). Cortical thinning in this region may therefore be relevant to the frontal-executive and cognitive difficulties observed in PD.

This finding is broadly consistent with recent longitudinal evidence linking cortical integrity to cognitive performance and cognitive decline in PD. Sokołowski et al. (37) reported that longitudinal changes in cortical thickness in the inferior temporal and precentral gyri were associated with changes in cognitive performance. More recently, Ren et al. (59) studied 195 patients with PD with annual cognitive assessment over a 3-year follow-up and found that baseline global cortical thickness was positively associated with MoCA scores, while reduced cortical thickness predicted subsequent cognitive impairment and was associated with more rapid cognitive decline. Although these studies assessed global or different regional cortical measures, they support a broader relationship between cortical structural integrity and cognitive performance in PD. The present study adds regional evidence by identifying a specific association between medial superior frontal cortical thickness and global cognitive performance. Importantly, this association remained statistically significant after controlling for age, sex, and education level and after Bonferroni correction for seven exploratory imaging–MoCA correlation analyses.

No other associations between MoCA scores and cortical thickness in the PreCG.R or altered GCA measures reached the Bonferroni-corrected significance threshold. However, these null findings should not be interpreted as evidence of an absence of clinical relevance, because the study may have been underpowered to detect smaller associations and the MoCA may not adequately capture the specific functions represented by these regions and networks. Nevertheless, the observed association should be interpreted cautiously. MoCA is a global cognitive screening instrument and may not be sensitive to specific cognitive domains related to frontal-executive, visuospatial, temporal, or sensorimotor network alterations (60, 61).

Nevertheless, the observed SFGmed.R–MoCA association should be interpreted cautiously. Unlike the longitudinal studies described above, the present study was cross-sectional and therefore cannot determine whether SFGmed.R thinning precedes or predicts cognitive decline. In addition, the observed association does not establish that cortical thinning in the SFGmed.R causes cognitive decline. Therefore, the association between SFGmed.R cortical thickness and cognitive performance should be considered exploratory and requires validation in independent and longitudinal cohorts using domain-specific neuropsychological assessments.

The present findings have several potential implications and may inform future research. First, reduced cortical thickness in the SFGmed.R and PreCG.R supports the involvement of frontal-executive and motor cortical systems in PD. The significant positive association between SFGmed.R cortical thickness and MoCA scores further suggests that structural integrity of the medial superior frontal cortex may be relevant to cognitive performance in patients with PD. Second, the abnormal GCA patterns indicate that structurally vulnerable cortical regions are associated with altered directional information flow involving temporal, visual, parietal, and olfactory-related networks. The involvement of an olfactory-related region is noteworthy given the high prevalence of olfactory dysfunction in PD. However, in the absence of objective olfactory testing, the clinical relevance of this finding remains speculative. Third, these structurally and functionally abnormal regions may serve as candidate imaging features for hypothesis generation and future validation. Their potential value as biomarkers of cognitive impairment, disease progression, or PD heterogeneity requires evaluation in larger longitudinal cohorts with external validation (58). However, the present findings, particularly the association between SFGmed.R cortical thickness and MoCA scores, should be regarded as exploratory and require validation in larger, longitudinal, and independent cohorts.

Several limitations should be acknowledged. First, the cross-sectional design precludes determination of the temporal relationships among cortical thinning, altered directed connectivity, and cognitive decline. Moreover, although GCA characterizes directional predictability between BOLD time series, it does not establish direct neural causality and may be influenced by limited temporal resolution and regional hemodynamic variability (47). Second, although disease duration and H&Y stage were available and descriptively characterized, they were not incorporated as covariates in the imaging analyses. Other potentially relevant clinical factors, including medication status, motor subtype, symptom laterality, cognitive reserve, and non-motor symptom burden, were not fully accounted for. Third, MoCA was the only cognitive measure used, and visual, olfactory, gait, and other domain-specific functions were not directly assessed. Consequently, the functional interpretations of the identified connectivity alterations remain exploratory. Fourth, the cortical clusters were identified from the same sample and subsequently used as seeds for GCA and as regions of interest for clinical correlation analyses. This data-driven procedure may introduce selection bias and inflate effect-size estimates. Fifth, the single-center sample, which included hospitalized patients with PD, may limit generalizability. Finally, although Bonferroni correction was applied to the exploratory imaging–MoCA correlations, the multiple seed-direction analyses and the data-driven nature of the study may still increase the risk of false-positive findings. Independent replication is therefore warranted. Independent replication, longitudinal follow-up, domain-specific behavioral assessment, and externally defined regions of interest are required to establish the robustness and clinical relevance of these findings.

In conclusion, the present study demonstrated that patients with PD exhibit cortical thinning in the right medial superior frontal gyrus and right precentral gyrus, and that these structurally abnormal regions show altered directional effective connectivity with temporal, visual, parietal, and olfactory-related regions. These findings suggest that PD-related cortical structural abnormalities are not isolated regional changes, but may be embedded within broader directional network dysfunction involving both motor and non-motor systems. This structural–effective connectivity framework may provide additional insight into the neural network basis of PD and may inform future studies on disease heterogeneity and imaging biomarkers.

## Data Availability

The raw data supporting the conclusions of this article will be made available by the authors, without undue reservation.
